# Cleft lip and palate genetics and application in early embryological development

**DOI:** 10.4103/0970-0358.57185

**Published:** 2009-10

**Authors:** Wenli Yu, Maria Serrano, Symone San Miguel, L. Bruno Ruest, Kathy K.H. Svoboda

**Affiliations:** Department of Biomedical Sciences, Texas A&M Health Science Center, Baylor College of Dentistry, Dallas, TX 75246

**Keywords:** Cleft lip/palate, Human/murine palate genetics, Palate development

## Abstract

The development of the head involves the interaction of several cell populations and coordination of cell signalling pathways, which when disrupted can cause defects such as facial clefts. This review concentrates on genetic contributions to facial clefts with and without cleft palate (CP). An overview of early palatal development with emphasis on muscle and bone development is blended with the effects of environmental insults and known genetic mutations that impact human palatal development. An extensive table of known genes in syndromic and non-syndromic CP, with or without cleft lip (CL), is provided. We have also included some genes that have been identified in environmental risk factors for CP/L. We include primary and review references on this topic.

## INTRODUCTION

Disturbances at any stage during palate development, e.g., defective palatal shelf growth, failed or delayed elevation, and blocked fusion, can result in cleft palate (CP)[1,2] with or without cleft lip (CL/P). As one of the most common congenital cranio-facial defects, CL/P occurs in approximately 1 per 750 live births in the United States[2,3] Clefts occur more frequently among Asians (about 1:400) and certain American Indians than Europeans or European descendants. Clefts are relatively less common among Africans and African Americans (about 1:1500).[4] Cleft lip and palate may not be life-threatening but many functions such as feeding, digestion, speech, middle-ear ventilation, hearing, respiration and facial and dental development can be disturbed because of the structures involved. These problems can also cause emotional, psycho-social, and educational difficulties. In addition, CP is an economic burden.

The aetiology of cleft lip with or without palate (CL/P) is theorized to be a combination of factors associated with genes and environment.[5,6] The advent of gene targeting technology and basic conventional techniques using animal models has led to the identification of genes associated with known and unknown etiologic factors. Characterization of the genomic sequences will greatly impact regulation of gene networks and pinpoint any variations in the different stages of craniofacial morphogenesis. In this article, emphasis is placed on different genes associated with the classifications of CL/P into syndromic [Table 1] and nonsyndromic [Table 2]. Each classification plays a significant role in understanding the molecular and genetic mechanisms affecting these types of craniofacial defects.[7–9] In addition to known genes there is strong evidence that several environmental factors (e.g., alcohol consumption, tobacco, and anti-convulsants) increase the risk of CL/P.[10,11] In contrast, several studies have shown that folic acid may have a protective effect on CL/P and neural tube defects.[12–16] Recent data from the National Birth Defect Prevention Network have indicated a decrease in neural tube defects from 5/10,000 to less than 2/10,000 after the fortification of the food supply with folic acid, indicating that this vitamin and the proteins that facilitate the uptake and metabolism of folic acid may be candidate genes in cranio-facial development.[14,17–20]

**Table 1:** Syndromic genes associated with cleft lip and palate

*Syndrome*	*Clinical Features*	*Genes*	*Reference*
Apert Syndrome (AS)	AD; high arched palate, bifid uvula, and cleft palate.	FGFR2	6, 114–118
Bamforth-Lazarus	AR; hypothyroidism, athyroidal, CPO, choanal atresia, spiky hair.	FOXE1	6, 119, 120
Syndrome (BLS)			
Branchio-oculo facial syndrome (BOFS)	AD; pseudocleft of the upper lip resembling a poorly repaired cleft lip.	TFAP2A	6, 121
Down syndrome (DS)	Macroglossia, microstomia, atlantoaxial subluxation	duplication of portion of chromosome 21	122
Ectrodactyly-ectodermal dysplasia-cleft syndrome (EEC)	AD; triad of ectrodactyly, ectodermal dysplasia, and facial clefting.	P63	6, 123, 124
Fetal alcohol syndrome (FAS)	Disorder characterized by a pattern of minor facial anomalies, prenatal and postnatal growth retardation.	alcohol dehydrogenase 1B (ADH1B)	125-128
Goldenhar syndrome (GS)	Oculo auricular vertebral dysplasia; AD; incomplete development of the ear, nose, soft palate, lip, mandible.	Pericentric inversion of chromosome 9	129, 130
Hereditary lymphoedema-distichiasis syndrome (HLD)	AD; lymphedema of the limbs, double rows of eyelashes, cardiac defects, and cleft palate.	FOXC mutations	131
Kallmann Syndrome (KS)	AR disorder; hypogonadotropic hypogonadism and anosmia	FGFR1 mutations	6, 132, 133
Margarita Island ectodermal dysplasia (ED4)	AR; unusual facies, dental anomalies, syndactyly, and cleft lip/cleft palate.	PVRL1 (nectin-1) mutation	6, 134
Pierre Robin	AD; triad of micrognathia, glossoptosis, and cleft palate.	Loci 2q24.1-33.3, 4q32qter, 11q2123.1, and 17q2124.325.1.	135,136
Sequence (PRS)			
Smith–Lemli-Opitz	AR; defects in cholesterol biosynthesis, growth retardation, dysmorphic facial features including CLP/ CPO, postaxial polydactyly	DHCR	6, 137, 138
Syndrome (SLMOS)			
Stickler Syndrome (SS)			
	AD; midface hypoplasia, micrognathia, Pierre Robin sequence, retinal detachment and early cataracts deafness, hypermobility of joints.	Col11A1, Col11A2, Col2A1	139,140
Treacher Collins (TC)	AD; craniofacial deformities such as downward slanting eyes, micrognathia, conductive hearing loss, underdeveloped zygoma.	Mutation in TCOF1 gene at chromosome 5q32-q33.1	141, 142
van der Woude syndrome (VDWS)	AD; cleft lip palate, distinctive pits of the lower lips, or both.	IRF 6 (interferon regulatory factor 6) mutations	6, 143
Velocardiofacial	AD; cleft palate, heart defects, abnormal facial structure, and learning problems.	Chromosome 22q11 microdeletion	144, 145
Syndrome (VCFS)			
Unnamed syndrome	CL/P and hereditary diffuse gastric cancer	CDH1	72
Unnamed syndrome	Chromodomain helicase DNA-binding proteins; CL/P in Charge syndrome	CHD7	146, 147
Unnamed syndrome	Bilateral CL/P, colobomas of the optic nerve and retina, agenesis of the corpus callosum. Dysphagia, reduced Oesophgeal peristalsis	PAX 9	6, 148
Unnamed syndrome	X-linked mental retardation and CL/P	PH8	6, 149
Unnamed syndrome	Holoprosencephaly 7, a spectrum of forebrain and midline anomalies and midline CL	PTCH	6, 137, 150, 151
Unnamed syndrome	CPO, craniofacial anomalies, osteoporosis, and cognitive defects	SATB2	6, 152
Unnamed syndrome	Holoprosencephaly, a spectrum of anomalies ranging from severe (cyclopia) to subtle midline asymmetries. CL/P part of the spectrum	SHH	6, 137
Unnamed syndrome	Anomalies with most features of DiGeorge/velocardiofacial syndromes: CPO, thymus and parathyroid gland hypoplasia, vertebra, facial and cardiac outflow anomalies.	TBX1	6, 153
Unnamed syndrome	X-linked CPO and ankyloglossia	TBX22	6, 51, 52
Unnamed syndrome	Cardiovascular, craniofacial, skeletal, and cognitive alterations, bifid uvula and or/CPO	TGF Beta receptor	6, 154

**Table 2:** Non-syndromic genes: interaction effects of genes and environmental risk factors on oral clefts

*Gene*	*Functional Role*	*Risk Factor*	*Reference*
Cytochrome P450 Proteins (CYP) CYPIA1, CYPIA2, CYPIB1 CYP2E1	Highly polymorphic, having multiple functional alleles; Role in detoxification; metabolism of endogenous morphogens in the developing foetus.	Negative gene-smoking interaction effect	155-157
Epoxide Hydrolase (EPHX)	Class of proteins that catalyze the hydration of chemically reactive epoxides into their corresponding dihydrodiol products.		
EPHX	Plays an important role in both the bioactivation and detoxification of exogenous chemicals such as PAHs, which are present in cigarette smoke.	Negative gene-smoking interaction effect	155, 158
EPHX1 Y113H	Variant of EPHX 1 found in the foetus and maternal smoking.	Positive gene-smoking interaction effect	28, 159
Glutathione Transferase Gene Family (GST)	Families of dimeric phase II enzymes that catalyze the conjugation of reduced glutathione with electrophilic groups of a wide variety of environmental agents.		
GSTM1	Major gene detoxifying PAHs and widely studied in many disorders and cancers.	Negative gene-smoking interaction effect	160, 161
GSTT1	Expressed in a variety of tissues/organs such as erythrocytes, lung, kidney, brain, skeletal muscles, heart, and small intestine; elevated expression profile at the craniofacial regions during embryonic development.	Positive gene-smoking interaction effect	162, 28, 157, 159
GSTP1	Major gene detoxifying PAHS; involvement in variety of disorders and cancers. Major enzyme involved in the inactivation of cigarette smoker's metabolites; most important isoform at the embryonic and early foetal developmental stages.	Positive gene-smoking interaction effect	163, 28, 159
GST A4 / GSTM3	Two other types of GST gene family members.	Positive gene-smoking interaction effect	28, 159
Hypoxia-Induced Factor-1 (HIF1A)	Mechanism by which maternal smoking may affect embryonic development due to the production of carbon monoxide, which interferes with oxygen transfer to the placenta, or nicotine, which constricts the uterine wall resulting in hypoxia.	Positive gene-smoking interaction effect	28, 159
Arylamine N-Acetyltransferase gene Family	N-conjugation of arylamine by the action of N-acetyltransferases (NATs), UDP glucoronosyltransferases (UGTs), or sulfotransferases (SULTS) produces nontoxic compounds.		
N-acetyltransferases1 (NAT 1)	Expressed in many tissues such as erythrocytes, bladder, lymphocytes, neural tissues, liver and intestines.	Negative gene-smoking interaction effect	19, 164, 165
N-acetyltransferases pseudogene, (NATP1)	Pseudogene identified, which is located at chromosome 8p23.1-8p21.3.		19, 164, 165
N-acetyltransferases2 (NAT 2)	Expressed in the liver and epithelial cells of the intestine.	Positive gene-smoking interaction effect	28, 157, 159
Methylenetetrahydrofolate reductase (MTHFR)	Metabolism of folate by reducing methylenetrahydrofolate, primary donor for methionine synthesis.	Positive gene-smoking interaction effect	166-172
MTHFRC677T	Variant of methylenetetrahydrofolate reductase.	Negative gene-smoking interaction effect	
OTHER METABOLIC GENES			
NAD(P)H quinine oxidoreductase (NQO1)	Flavoenzyme that catalyzes two electron reduction of quinine compounds to hydroquinone and is inducible by oxidative stress, dioxin, and PAHS found in cigarette smoke	Negative gene-smoking interaction effect	28, 159
SULT1A1	Catalyzes transfer of the sulfonate group from the active sulfate to a substrate to form the respective sulfate or sulfamate ester.	Negative gene-smoking interaction effect	28, 159
UDP glycosyltransferases (UGTs) UGT1A7 variant	Catalyzes conjugation reactions where hydrophobic chemicals are transformed into water-soluble compounds. Potential maternal effects on embryonic development.	Positive gene-smoking interaction effect	159, 173, 174
DEVELOPMENTAL GENES FOR ORAL CLEFTS			
Transforming Growth Factor A (TGF α)	Transmembrane protein expressed at the medial edge of the epithelium (MEE) of fusing palatal shelves. Its receptor epidermal growth factor (EGFR) is expressed in the degenerating MEE.	Positive gene-smoking interaction effect (smoking, alcohol drinking, vitamins)	175-177
Transforming growth Factor β-3 (TGF β3)	Regulator of many biological processes such as proliferation, differentiation, epithelial mesenchymal transformation and apoptosis.	Positive gene-smoking interaction effect (smoking, alcohol drinking)	81, 176, 178
Muscle Segment Homeobox1 (MSX1)	Transcriptional repressor important in craniofacial, limb, and nervous system development.	Positive gene-smoking interaction effect (smoking and alcohol drinking)	176, 179, 180
MSX2	Similar to MSX1; rare cause of isolated cleft lip with or without cleft palate.		179, 180
Acyl-CoA desaturase ACOD4	Pericentric inversion disrupts a gene (ACOD4) on chromosome 4q21 that codes for a novel acyl-CoA desaturase enzyme that occurs in a single two-generation family with CL.		181
Retinoic acid receptor (RAR)	Odds ratios for transmission of alleles at THRA1 were significant when ethnic group was included.	Negative gene-smoking interaction effect	176
CHD7	Chromodomain helicase DNA-binding proteins.		182
ESR1	Ligand-activated TF estrogen receptor.		183
FGF/ FGFR families FGF8 FGF3 FGF10 FGF18 FGFR1 FGFR2 FGFR3	Expressed during craniofacial development and can rarely harbor mutations that result in human clefting syndromes.		184
SPRY1/SPRY2	Loss of function mutations in FGFR1 cause a syndromic form of clefting.		185
TBX10	Ectopically expressed in dancer cleft lip and palate mutant mice.		185
GABRB3	β3 subunit of GABA receptor CL/P.		62, 186, 6
GLI2	Mutations in GLi2 cause holoprosencephaly-like features with cleft lip and palate.		185
ISGF3G	Similar to IRF6.		185
OTHER CANDIDATE GENES			
SKI, FOXE1, JAG2, LHX8	Rare causes of isolated cleft lip with or without cleft palate		185

This review will concentrate on genetic contributions to facial clefts with/without cleft palate. We will begin with an overview of early palatal development, concentrate on muscle and bone development, and incorporate the effects of environmental insults and known genetic mutations that impact human palatal development.

## EMBRYONIC PALATE DEVELOPMENT

**Figure 1:**
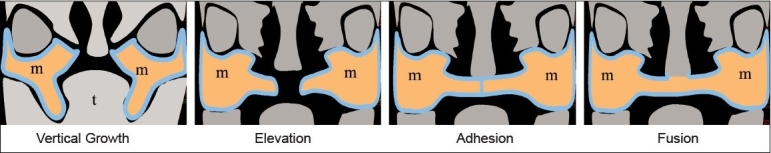
Schematic drawing showing coronal view of a normal palate shelf and key stages of mouse palatal development. At E12-E13 days in the mouse gestation, the palatal shelves grow downward along the tongue (t). At E13-E13.5 days, the palatal shelves become elevated above the tongue. At E14.5, the palatal shelves adhere to each other in the midline. After E15.5 days, the MES completely degrades, and the palate fuses

The palatal structures are composed of the cranial neural crest (CNC)-derived mesenchyme and pharyngeal ectoderm.[21–24] Epithelia that cover the palatal shelves are regionally divided into oral, nasal and medial edge epithelia (MEE). The nasal and oral epithelia differentiate into pseudo-stratified and squamous epithelia, whereas MEE is removed from the fusion line [Figure 1].

The secondary palate originates as an outgrowth of the maxillary prominences at approximately embryonic day 11.5 in the mouse (E11.5-m) [Figure 1] and post coital six weeks in humans (p.c.6wk-h). The palate shelves initially grow vertically along the sides of the tongue (E13.5-m; p.c.7wk-h) and then rise above the tongue as the latter drops in the oral cavity due to the forward and downward growth of the mandible (E14.0-m; p.c.8wks-h). With continued growth, the shelves appose at the midline (E14.5-m; p.c.10wks-h) and eventually fuse (E15.5-m; p.c.13wk-h).[25] Numerous genes similar in mice[26] and humans[25,27,28] are expressed [Table 1] during palatal development.

**Figure 2:**
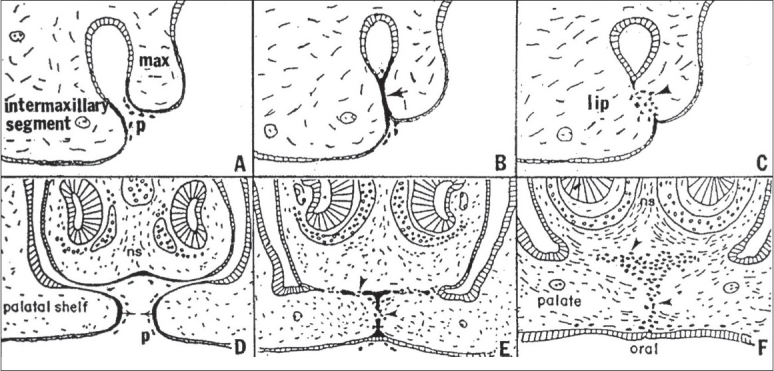
Comparison of the morphogenesis of the upper lip (A-C) with that of the palate (D-F). After the bilateral maxillary processes (max) fuse externally with the inter-maxillary segment, the resulting epithelial seam (arrow, B) gives rise to mesenchyme (arrowhead, C) to produce a confluent lip. At a later time, the palatal shelves arising internally from the maxillary processes fuse with each other (arrows, D) and with the nasal septum (ns) above them, creating an epithelial seam that transforms to mesenchyme (arrowheads, E) to produce the confluent palate (arrowheads, F). p, sloughed periderm cells. Reprinted with permission.[44]

During fusion the epithelium covering the tip of the opposing palatal shelves, adheres, intercalates and thins into a single-layer midline epithelial seam (MES).[23] The disintegration of this seam results in the confluence of the palatal mesenchyme. Tremendous interest has arisen in cellular mechanisms underlying MES degradation. Epithelial-mesenchymal transition (EMT) is one of the proposed models that regulates medial edge epithelial (MEE) cell fate.[23,29–36] However, other mechanisms have been proposed, such as apoptosis,[37–40] in which all MEE cells are theorized to die during fusion. Alternatively, it is hypothesized by some researchers that MES cells disappear by migrating from the midline towards the nasal and oral epithelia.[41,42] Other investigators postulate that all events, including apoptosis, migration and EMT, may occur.[23,39,43] Interestingly, the fusion of the external surface of the bilateral maxillary processes with the naso-frontal prominence in the chick is similar to palatal fusion [Figure 2].[44] The outer periderm layer dies through apoptosis, and the lateral edge epithelium of the inter-maxillary segment of the naso-frontal process fuses with the medial edge epithelium of the external maxillary process to form a seam that transitions to a confluent mesenchyme [Figure 2].[44] Evidence supporting these theories, especially those involving EMT and apoptosis, will be presented and further discussed.

## MOLECULAR SIGNALLING EVENTS IN EMBRYONIC PALATAL DEVELOPMENT

As stated above, cleft palate with or without cleft lip is a complex trait caused by a combination of multiple genes and environmental factors.[5] Palatal shelf development defects will be divided into five categories for the purpose of this review:

### Failure of palatal shelf formation

The failure of the palatal shelf formation is a rare severe defect. Recent studies have identified several molecular networks operating between the palatal shelf epithelium and mesenchyme during different steps of palatogenesis. These networks include signalling molecules and growth factors such as sonic hedgehog (Shh), members of the transforming growth factor β (TGfβ) super family, including bone morphogenetic proteins (Bmps) and Tgfβs, fibroblast growth factors (Fgfs) and their receptors (FgfR), effectors and targets.[25] Studies addressing the role of Fgf signalling during early palatal development by analyzing Fgf10 and FgfR2b mutants found altered cell proliferation within both mesenchyme and epithelium in the palatal shelves and increased apoptosis within the epithelium. It was reported that Fgf10 and FgfR2b mutations affected the initial development of palatal shelves, and the mouse pups had complete CP.[45] By signalling via its receptor, FgfR2b, in the palatal shelf epithelium, the mesenchymal derived Fgf10 supports epithelial proliferation and survival and also induces the expression of Shh within the epithelium. Shh, in turn, signals to the mesenchyme and stimulates cell proliferation.

In general, signalling activities are subject to tight spatio-temporal control, and, in many instances, too much or too little control is detrimental to the developing organ. This situation is well illustrated in anomalies caused by de-regulated hedgehog (hh) and Fgf signalling.[46,47] While Fgf10/FgfR2b activity plays a crucial role during palatogenesis, it appears to be subject to the tight spatio-temporal regulation shown in mice lacking *Shox2. Shox2* mutant mice develop a very rare type of CP that may also be found in humans[48] the soft palate is intact, whereas the hard palate has a cleft. Abnormal proliferation and apoptosis are theorized to be the cause of the cleft. Surprisingly, a number of protagonists implicated in palatogenesis, including *Msx1, Bmp4, Pax9, Lhx8, Osr2*, *Tgfβ3* and *Jag 2*, were expressed normally.[48] In contrast, Fgf10 and Fgfr2b were expressed at ectopic sites within the mesenchyme of the Shox2 mutant mice.[49] These studies emphasize the importance of the precise timing and determination of sites of signalling activities necessary for normal development. Mutation of activin-βA causes a severe facial primordial development defect, which may be responsible for the retardation of palatal shelf development and complete cleft palate. In addition, other genes, including *Msx1, Lhx8, Shox2* and *Osr2*, assume important roles in the palatal shelf growth. The targeted mutation of these genes in mice generates CP, indicating the intrinsic requirement of these factors during palatogenesis.[49]

### Fusion of the palatal shelf with the tongue or mandible

Under normal conditions, palatal shelves do not fuse with other oral structures. However, in mice that do not express Fgf10, the palatal shelf epithelium fuses with the tongue and mandible.[45] The loss of function mutations of Fgf10 results in anterior palatal shelf fusion with the tongue, whereas the middle and posterior palatal shelf regions adhere to the mandible, thus preventing the elevation of the palatal shelf.[50] There is a severe reduction of the expression of *Jagged 2* (*Jag2*), thereby encoding a ligand for the Notch family receptors and ectopic Tgfβ3 production in the nasal epithelia of these mice. The analysis of *Jag2* mutant embryos indicates that Jag2-Notch signalling prevents inappropriate palatal shelf adhesion to other oral epithelia through the control of oral epithelial differentiation. Another gene has also been associated with inappropriate adhesions. Mutations in TBX22 have been reported in families with X-linked cleft palate and ankyloglossia.[51–53]*Tbx22* is expressed in the developing palate and tongue in mice, suggesting an important role in regulating tongue and palate development.

### Failure of palatal elevation

Palatal shelf elevation is a rapid movement triggered by both intrinsic forces within the palatal shelves proper and by influences from other craniofacial and oral structures, including the movement of the tongue, and growth of the cranium and mandible.[1,54] The role of the extra-cellular matrix in palatal shelf elevation has been supported by some studies and is presently accepted as an important determinant of palatal shelf elevation.[55,56] Those studies[1] suggested that a progressive differential accumulation of glycosaminoglycans, primarily hyaluran in the palatal shelves, plays a role in their elevation.[55,56] Hyaluronan is a highly charged glycosaminoglycan that retains high amounts of water, forming hydrated gels leading to the expansion of the extracellular matrix. Other constituents of the palatal shelves including collagen fibers, vascularization, and the epithelial covering; the polarized alignment of the mesenchyme cells may also contribute to the intrinsic elevation force of the PS. Mutations of *Pax9, Pitx1* or *Osr2* can lead to failed palatal shelf elevation and cleft palate defect.[57–60] The cellular defect is associated with the CNC-derived palatal mesenchyme, suggesting the important functions of these transcription factors in regulating the fate of the CNC cells during palatogenesis.

Early studies attributed a role to neuro-transmitters during palatal shelf elevation.[1] At present, it is widely accepted that neuro-transmitter γ-aminobutyric acid (GABA) regulates not only neuronal activities but also cell migration, survival, proliferation and differentiation of neuronal and non-neuronal cells.[61–63] Terratological studies in rodents showed that GABA or GABA agonists generate CP by inhibiting palatal shelf elevation, whereas GABA antagonists stimulate the process.[64] The implication of GABA in palate development was demonstrated by genetic studies of mice lacking the β3 subunit of the GABA receptor that developed CP without other craniofacial malformations.[65]

### Failure of palatal shelves to meet after elevation

Fusion of the opposing palatal shelves is an important step taking place through a sequence of events that includes the removal of the flat peridermal cells, contact and adhesion of the opposing MEE, which creates the MES, and the degeneration of the MES. The mesenchymal confluence thus forms at the midline.[22,23,34] Failure of shelf fusion is the most common type of cleft palate defect documented in animal studies. Mutations in *Msx1* and *Lhx8* and conditional inactivation of *Tgfbr2* in CNC cells or *Shh* in the epithelium all result in retarded palatal shelf development.[45]

In many transgenic animals, the palatal shelves fail to meet at the midline because of hindrance by the tongue. This is usually associated with cases when the lower jaw does not move forward and downward during development, keeping the tongue between the palatal shelves. These secondary defects were evident in the *Hand2* mutant mice in which the enhancer driving the expression of the gene in the pharyngeal arches was inactivated by targeted mutagenesis.[66] In these mice, the mandible did not grow properly, blocking the descent of the tongue, thus hindering palate fusion.[66,67]

### Persistence of middle edge epithelium

Adhesion of the opposing MEE is an important event in both human and mouse embryos.[21,27,34,44,68] E-cadherin is expressed in the epithelia covering the fronto-nasal and medial nasal processes as well as during the different stages of palate development, including the epithelial islands, remnants of the MES.[69–71] Mutations of *CDH1*/E cadherin, which deletes the extracellular cadherin repeat domains required for cell-cell adhesion, have recently been associated with CL/P in families with hereditary diffuse cancer.[72] E-cadherins are known to form dimers, indicating that the mutant proteins may have trans-dominant negative effects over the normal proteins.[72]

Extensive efforts have been made to elucidate the role of Tgfβ3 during palatal fusion.[73–76] Adhesion of the MEE upon palatal shelf contact is a necessary step for fusion. TGfb3 is expressed in the MEE before and during fusion, and mediates MEE adhesion of the opposing palatal shelves through filopodia. E-cadherin is required for fusion, whereas filopodia seem to be crucial for proper alignment and guidance of cell sheets that are fated to fuse, but not for fusion itself.[77] Tgfβ3 is implicated in controlling the re-modelling of the extracellular matrix through regulation of the expression of the matrix metaloproteinases (Mmps) Mmp13, Mmp2 and the tissue inhibitor of metaloproteinase-2 (Timp).[78] Tgfβ3 signalling functions in the MEE by mediating the epithelial-masenchymal interactions leading to tissue changes that regulate palatal fusion. For example, EMT of the MES has been proposed as the major mechanism underlying the disappearance of the MES to generate mesenchyme continuity, thus preventing palatal clefts.[34] The establishment of the concept of EMT as the prevailing mechanism of MES disappearance led to studies attributing roles to different molecules, including Tgfβ3, Lef1, Smad, RhoA, phosphatidylinositol 3-kinase (PI-3 kinase), Mmps Twist and Snail.[22,33,79] In *Tgf*β3 or *Egfr* mutant mice, there is an alteration of the fate of MEE cells.[80,81] In Tgfβ3 null mutant mice, MEE cells fail to undergo apoptosis and remain along the midline, preventing normal fusion.

## OSSIFICATION OF THE PALATE

Palatal fusion signals the start of the ossification process in the anterior two-thirds of the palate to form the hard palatal tissues. This process entails the successful fusion of the three embryonic structures - lateral edges of the primary palate with the two anterior edges of the secondary palate. This process requires the synchronization of shelf movements together with the growth and withdrawal of the tongue and growth of the mandible and head.[82] Any form of disruption during the formative stages results in a pathological cleft. The same is true when ossification occurs too early. *Sox9* is a gene controlling cartilage development and blocking the expression of *Runx2*, a transcription factor essential for osteoblast differentiation and bone formation associated with cleidocranial dysplasia. In *Sox9* mutant, *Runx2* expression is not repressed and ossification begins prematurely.[83] Since the palatal shelves are prematurely ossified, they cannot grow toward the midline and fail to fuse.

A wide range of studies on cranio-facial skeletal maturation has shown that the fusion of the palatal shelves along their length to form the mid-palatal (MP) suture occurs during the ossification of the maxillae and palatine bones before the mandibular condyle develops.[48,84,85] Ossification is observed where mesenchymal cells condense, the surrounding tissue vascularizes and the cells differentiate into osteoblasts that will form bone by mineral deposition. In this process, several growth and differentiation factors such as Bmps, core binding proteins (Cbf), Fgfs, and hedgehog (hh) proteins that interact with various signalling pathways to regulate the patterning of the undifferentiated mesenchyme, are involved. The Bmp-6 and the transcription factor Gli1 are also expressed during intra-membranous bone formation.[86,87] As in cranio-facial sutures, the MP and trans-palatal (TP) suture osteoblasts express Tgfβ1, 2 and 3, while the suture cells express primarily Tgfβ3.[88,89]

**Figure 3:**
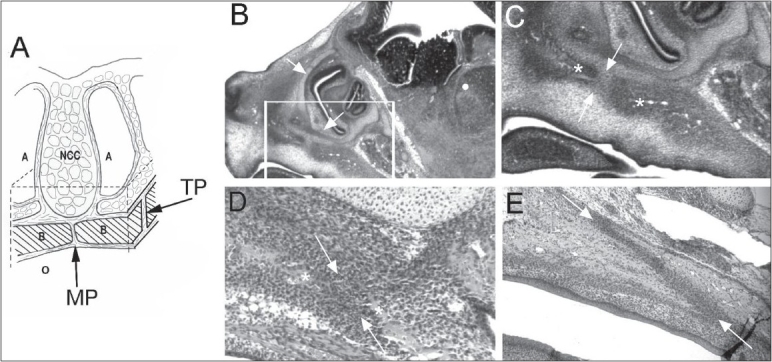
(A) Diagram showing the relationship between the NC cartilages and the transpalatal suture. Dotted lines indicate cut lines for removing the palate from the embryo and the NC cartilage from above the sutures. (B-E) Micrographs of parasagittal sections of foetal rat heads show the pre-natal development of TP sutures. (B) At E16, NC cartilages (arrows) can be seen directly above the presumptive TP suture region (in box). (C) High-power micrograph of the region in the box, showing the advancing palatal plate of the maxilla and horizontal plate of the palatal bone (asterisks) on either side of the presumptive TP suture (between arrows). (D) At E18, the advancing bone fronts (asterisks) begin to overlap one another, creating a highly cellular suture blastema (between arrows). (E) By E20, an elongated TP suture (between arrows) continues to form as the bone fronts proceed to overlap one another. A, airway; B, shelves of maxillary bones; MP, midpalatal suture; NCC, nasal capsular cartilage; O, oral cavity; TP, transpalatal suture. Reprinted with permission.[87]

It has been established that cranial sutures are the growth sites for the neuro-cranium and that the dura mater provides the signalling molecules to regulate suture patency.[90] The MP and TP sutures have different morphology, so they are not in contact with the dura mater. Opperman's group hypothesized that these facial sutures are growth centres[88,89] and that the nasal capsular cartilage produces signalling molecules to regulate the fusion of MP and TP sutures [Figure 3].[89] They found that the nasal cartilage maintained the TP sutures as growth sites in experiments on rat palatal organ cultures (E20) with or without nasal cartilage. They theorized that the nasal cartilage may regulate mid-facial growth.[89]

Animal models have been developed to understand the aetiology and pathogenesis of orofacial clefts and the mechanisms of normal palatal ossification. The application of cyclic forces is an effective mechanical stimulus for the regulation of osteogenesis and osteoclastogenesis in the sutural growth of neonatal rats.[91] The process of tissue response and regeneration in the palato-maxillary suture under tensile forces was examined histologically and with fluorescence. A cyst-like zone appeared in the conjuncture of the bony front and the sutural connective tissue at the early stage of sutural expansion with increased proliferating osteoblasts and fibroblasts. New bone was deposited along the nasal septum and the front of the cyst until the new bone front formed and the suture restored its original morphology.[92]

The approach of utilizing MP suture expansion in mice has provided new insights into mechanical stress modulation as an important factor for the skeletal remodelling of bones and cartilage. The expansive force across the MP suture promotes both bone resorption through the activation of osteoclasts and bone formation through the increased proliferation and differentiation of the periosteal cells.[93] Similarly, the use of orthodontic wire expansion in growing rats showed that secondary cartilage can undergo chondrogenic and osteogenic differentiation in the maxillary arch. Interestingly, these induced changes were attributed to the alteration of the differentiation pathway of progenitor cells from chondroblastic to osteoblastic, in which many sutures temporarily form secondary cartilage during early development. Histological observations at days 7, 10, and 14 indicated that intra-membranous bone formation, which is partially recognized as mature bone,[94] occurred at the boundary between the pre-cartilaginous and cartilaginous cell layers where the calcified matrix was positive for osteocalcin antibody. The cellular events taking place at the MP suture cartilage in rat models as a result of expansion force have been observed as endochondral bone formation at the boundary between the maxillary bone and cartilage, whereas intra-membranous osteogenesis has appeared at the internal side of the cartilaginous layer.[95] To stimulate new bone formation in defective tissues, rat organ cultures with distracted palatal sutures were treated with Bmp-7 and Nell-1 for 8 days *in vitro*. The presence of Nell-1 increased chondrocyte hypertrophy and endochondral bone formation while Bmp-7 enhanced both chondrocyte proliferation and differentiation in the distracted palates of four-week-old male rats. This study indicates that Nell-1 is involved in the rapid osteoblast differentiation in palate sutures.[96] In another study, the application of TGF-β1 during the early stages of rat MP expansion induced rapid bone formation at the suture site.[97]

## ORAL AND PALATAL MUSCULATURE AND RELATED DEFORMITIES

Overt CL/P encompasses a broad spectrum of defects, ranging from so-called microform clefts to complete unilateral or bilateral clefts of the lip and palate. The orbicularis oris (OO) muscle consists of numerous differently oriented strata of muscular fibres that surround the orifice of the mouth. At approximately seven weeks post-conception (p.c.) in humans, the two maxillary prominences fuse with the medial nasal prominence; however, lip fusion is not complete until the epithelial seam disappears through EMT and/or apoptosis[82] [Figure 2A–C]. By eight weeks p.c., a dense, continuous band of mesenchymal cells corresponding to the future OO muscle can be seen, with discernible OO muscle fibers present by 12 weeks.[98,104] The complete OO muscle architecture forms by 16 weeks. Any delay in fusion may result in sub-epithelial OO defects, such as the altered migration of the mesenchymal cells. Sub-epithelial (non-visible) defects of the orbicularis oris muscle represent the mildest form of cleft lip, and such defects are part of the phenotypic spectrum of CL/P. This defect usually is visualized as a ridge of tissue resembling a scar on the upper lip along the philtrum.[98]

Histological studies have demonstrated that such defects extend to the muscle fibres of the superior OO muscle. A method using high-resolution ultrasonography (USG) was developed to visualize the OO muscle non-invasively.[99] Significant differences in the defects of the OO are found in the first-degree relatives of CL/P individuals and controls. The OO muscle defect detected by ultrasound is consistent with the histological examination of cadavers.[99] Interestingly, the Bmp4 knockout mouse model shows bilateral cleft lip at E14.5, although this condition occurs at a rate of 22% after birth,[100] suggesting the initial cleft lip is rescued or healed *in utero*, leaving only the subepithelial OO defect. Potential mutations in BMP4 were found in two individuals with OO defects and none in the controls.[101] The strong evidence that OO discontinuities are indeed part of the phenotypic spectrum of CL/P provides an important clue for the clinical recurrence risk estimation for families with members affected with CL/P.

The mildest form of CP is termed a “submucosal cleft palate,” described as a bifid uvula, palatal muscle diastasis and a notch in the posterior surface of the hard palate.[102] Defects in the nasopharyngeal anatomy and/or physiology may lead to velopharyngeal incompetence (VPI). Although most VPI is caused by CP, the population prevalence of VPI due to other causes is estimated to be approximately 2.5%.[103] In such cases, VPI may be caused by submucosal muscular defects of the levator veli palatini or musculus uvulae. Most of the soft palate muscles are derived from myotome cells, which first invade pharyngeal arch 4 and then migrate to the palate, carrying their innervations from the vagus nerve. One muscle (tensor veli palatini) is derived from myotome cells that first invade arch 1 and are innervated by the trigeminal nerve.[104] In the mouse, the tensor veli palatine, levator veli palatini, medial pterygoid, and lateral pterygoid muscles are identified as myogenic fields as early as gestational day 15. The palatoglossus, palatopharyngeus, and musculus uvulae, however, are not clearly visible.[105] In principle, the presence of these anatomical features in unaffected individuals may signify an elevated risk for producing clefts in offspring.[106]

## SUMO MODIFICATION OF SIGNALLING PATHWAYS IN PALATOGENESIS

The molecular understanding of NS CL/P is further complicated when one considers that large differences in penetrance often occur when the same mutations are placed on different mouse strains, indicating a potential role for both genetic and/or environmental modifiers in the pathogenesis of CL/P. Several lines of evidence point to the involvement of the small ubiquitin-like modifier (SUMO) posttranslational modification machinery.[107] A surprisingly specific role in oro-facial development has been revealed for protein modification by the SUMO, which might hint at a possible interaction with environmental factors. Small ubiquitin-related modifiers belong to the ubiquitin-related protein family, and SUMO proteins are ubiquitously expressed throughout the eukaryotic kingdom.[108] SUMO1 shows strong expression in the MEE of the secondary palate.[109] A translocation breakpoint interrupting SUMO1 was found in a patient with CLP.[109] The causative nature of the translocation defect has been confirmed in SUMO1-deficient mice having a distinct CP phenotype.[109] Furthermore, it was recently shown that mutations in TBX22 have a profound effect on its ability to be “sumoylated,” which is at least partially responsible for its loss of function.[110] Other SUMO targets include Smad4, Msx1, p63, Pax9, Eya1 and FGF signalling.[107] It seems likely that some of these factors may manifest through the disturbance of the SUMO pathway. De-stabilizing the normal balance of expression and activity for genes such as TBX22, MSX1, SATB2, and P63 during early pregnancy is likely to provide a high-risk environment for the occurrence of CL/P. Elucidating the relationship among environmental factors, the SUMO pathway, and the networks of craniofacial genes influenced by this post-transcriptional modification may be crucial to our understanding of the idiopathic forms of oro-facial clefts.

## A-P GRADIENT OF MOLECULAR SIGNALLING IN PALATAL DEVELOPMENT

Multiple genes are critical for the development of the anterior region of the palate. *Msx1, Bmp4, Bmp2, Fgf10*, and *Shox2* have restricted expression patterns in the anterior region of the palate.[45] In addition to the differential gene expression patterns along the A-P axis of the developing palate, there is also mesenchymal heterogeneity between the medial and lateral regions of the palatal shelf. The odd, skipped related genes *Osr1* and *Osr2* are expressed in a medial-lateral gradient in the palatal shelf. The mutation of the *Osr2* gene results in the compromised development of the medial aspect of the palatal shelf and retards palatal shelf elevation.[60,111] The expression of *Fgfr2* is focused on the medial aspect of the developing palatal shelf, suggesting a possible functional significance in regulating its development and elevation.

An important discovery has been the confirmation of genetic heterogeneity along the anterior-posterior and medial-lateral axes of the developing palate.[48] This heterogeneity may provide a differential regulatory mechanism for the fusion of the anterior vs. posterior region of the palate. MEE cells undergo apoptosis at different times during palatal fusion. It has been shown that the apoptosis of MEE cells is triggered by palatal shelf contact in the anterior region, whereas it is initiated before any contact between the opposing shelves in the posterior region.[38] This difference may be the result of dissimilar molecular signals in the palatal mesenchyme along the anteroposterior axis that instruct different fates to the palatal epithelium.[112] Recent studies have demonstrated that constant and reciprocal interactions between palatal epithelium and CNC-derived mesenchyme are responsible for setting up this genetic heterogeneity along the AP axis and are crucial for normal palatal development and fusion.[25,45,113] The specific gene expression patterns in the posterior region of the palatal mesenchyme are less understood. *Fgfr2* is expressed in the epithelium, and the CNC-derived mesenchyme is found in the middle and posterior palate. FGF8 signalling selectively induces the expression of *Pax9* in the posterior region of the palatal mesenchyme. The loss of *Pax9* results in a palatal shelf development defect and a cleft palate[48,58]

## CONCLUSION

It is clear from this and other review of literature that CL/P is caused by many factors, including both genes and environment. Gene targeting technology and basic conventional techniques using animal models led to the identification of genes associated with known and unknown aetiologic factors. In some cases, the human gene deficiency was identified first and replicated in an animal model, but in other cases, animal models led the way to understand gene/environment interactions. It is also clear from this extensive list of possible contributing genes that the molecular and cellular interactions associated with CL/P are not all understood. Fortunately, some subclinical changes in facial features may lead to a greater understanding of the gene/environment interactions in cranio-facial development.
